# A randomized clinical trial of fibromyalgia treatment with acupuncture compared with fluoxetine

**Published:** 2012-10-30

**Authors:** M J Hadianfard, M Hosseinzadeh Parizi

**Affiliations:** 1 Associate Professor, Department of physical medicine and rehabilitation, Shiraz University of Medical Sciences, Shiraz, Iran; 2 Resident, Department of physical medicine and rehabilitation, Shiraz University of Medical Sciences, Shiraz, Iran

**Keywords:** Randomized trial, Acupuncture, Fibromyalgia (FMS)

## Abstract

**Background:**

To evaluate the effectiveness of acupuncture and compare it with fluoxetine in treatment of fibromyalgia syndrome (FMS).

**Methods:**

We conducted a prospective, randomized clinical trial. Fifteen patients were treated with acupuncture and compared with a control group (n=15) of patients who received fluoxetine. Visual analogue scale, Fibromyalgia Impact Questionnaire (FIQ) and determined number of tender points were used as outcome measurements.

**Results:**

After four weeks, the acupuncture group was significantly better than the control group in the number of tender points. Total fibromyalgia symptoms were significantly improved in the acupuncture group compared with the control group during the study period (P= 0.01). The largest difference in mean FIQ total scores was observed at 4 weeks (42.2 VS. 34.8 in the control and acupuncture groups, respectively; P= 0.007). At the end of one year of the follow up, patients who received acupuncture were significantly better than the control group in all measures. Fatigue and anxiety were the most significantly improved symptoms during the follow up period.

**Discussion:**

Acupuncture significantly improved pain and symptoms of fibromyalgia. Also, we found that acupuncture did not have any side effect and was tolerable.

## Introduction

Fibromyalgia syndrome (FMS) is a diffuse musculoskeletal pain syndrome with multiple tender points that are widely and symmetrically distributed throughout the body and lasting longer than 3 months. It is associated with chronic fatigue, cognitive dysfunction, sleep disorder, morning stiffness, anxiety and depression. For most patients, these chronic symptoms negatively affect their quality of life and functional performance (1-3). The etiology of FMS remains unknown (4,5).

In Iran, fibromyalgia is estimated to affect 4% of the population (6). This prevalence is similar with other countries (7,8). Despite increasing interest in and understanding of this complex syndrome, effective and specific treatment is still not available, and there are no treatment approved by US Food and Drug Administration (9,10). Usual treatment typically involves the chronic use of medications for pain control. Low doses of antidepressants (11), physical activity such as aerobic exercise (12), relaxation techniques (13), behavioral therapies (14,15), and nutritional supplementation (16) were mentioned.

Acupuncture is one of the oldest forms of therapy which is widely practiced these days, mainly to relieve pain caused by a variety of ailments (17-20). A consensus statement from the US National Institutes of Health (NIH) concluded that acupuncture may be useful as an adjunct treatment or may be an acceptable alternative treatment in a comprehensive management program for fibromyalgia patients (21). In Iran also there has recently been an increased interest in acupuncture.

The objective of this study was to evaluate the benefits of acupuncture compared with SSRI antidepressants (fluoxetine) in patients with FMS. The aim of this study was to assess the efficacy of an alternative therapeutic approach.

## Materials and Methods

### Patients

We included patients diagnosed with FMS according to the 1990 American College of Rheumatology (ACR) classification criteria (22). To be included, patients had to report moderate to severe pain intensity with visual analogue scale (VAS) more than 4.

Patients were excluded in the case of severe psychiatric disease, the presence of neurological deficits, rheumatologic disorders, cardiac disease and any other significant systemic disorders which might affect the results, and patients with any problems for which acupuncture or prescription of drug were contraindicated (such as glaucoma).

The patients fulfilled all inclusion criteria and agreed to participate in the trial. The patients were informed about the treatment protocol and admitted to the study only after giving their written, informed consent. The study protocol was approved by the ethics committee of the university. Table 1 summarizes the patients’ baseline characteristics.

**Table 1:** Baseline clinical and demographic characteristics of patients with fibromyalgia by treatment group.

Characteristics	Acupuncture group	Fluoxetine group
Gender (female), n(%)	15 (100)	15 (100)
Mean age, years, mean (SD)	43.86 (7.9)	44.2 (10.8)
Marital status, n (%)		
Married or common-law	13 (86.66)	14 (93.33)
Single, divorced, widowed	2 (13.34)	1 (6.67)
Occupation, n (%)		
Housewife	13 (86.66)	11 (73.33)
Working	2 (13.34)	4 (26.67)
Pain duration,months, mean (SD)	82.8 (68.4)	79.6 (69.8)

### Interventions

The patients were randomly allocated to two groups: acupuncture and fluoxetine (control) group. Randomization was performed using a computer-generated random sequence of the numbers provided by the Hospital’s Informatics Departments. The randomization was conducted by one physician who was not involved in the inclusion or exclusion process.

The patients in the acupuncture group received two weeks of three sessions (weekly) lasting for 30 minutes in each session. The acupuncturist used disposable, sterilized, flexible stainless steel 0.25 × 40 millimeter needles. The classical acupuncture points employed were: ST-36, GB-34, RN-6, SP-6, LI-4, ST-44, BL-40, HT-7 and DU-20 (23). Needle penetration was 10–30 millimeter without extra rotational or manual stimulation after needle insertion. Depth of needle penetration was determined by the patient’s sensitivity until "chi" sensation was obtained. The inclination of the needle was 90º in all points.

Control group received 20 mg fluoxetine (Dr. Abidi, Tehran, Iran) orally every morning for 8 weeks. Two groups were seen by a physician at the beginning of the study and during the follow-up visits.

### Outcome assessment

The primary outcome measure was pain intensity evaluated using a VAS score (10 cm). Other outcomes were the number of tender points below 4kg/cm^2^ pressure (TPN) and the Fibromyalgia Impact Questionnaire (FIQ).

The patients rated their pain intensity using a VAS, where "zero" corresponds to no pain and "10" corresponds to the worst pain experienced by the patient. The number of tender points is a criterion for diagnosis of fibromyalgia. According to the ACR, there should be at least 11 of 18 tender point sites that are painful with digital palpation pressure of 4 kg. For functional and psychological outcome we used FIQ score (24). It is the sum of the 8 FIQ subscales (each on a 0- to 10- point scale) and 20 items. The first 11 items identify the patient’ function including shopping, laundry, meal preparation, dish washing, sweeping, bed preparing, walking 100 meters, working in the house garden, driving, and going up the stairs. Two items are the number of days that the patient missed work due to disease and the number of days he/she had a good feeling. The final seven items evaluate psychological statue including depression, anxiety, tiredness, stiffness, working problem, fatigue and pain severity. Patients who scored more had more severity of disease. The primary analysis involved comparing the FIQ total score between the acupuncture and fluoxetine group over time using a repeated-measures analysis of the variance model. An overall treatment effect was estimated after adjusting for time and baseline FIQ total score. Examining treatment effects at individual time using analysis of covariance complemented this analysis.

Outcome assessments were conducted at baseline, 2,4 and 8 weeks, and again after 12 months of the first evaluation. All evaluations were obtained by the study coordinator, who was blind to the group's assignment.

### Statistical analysis

This study was designed to have 80% power to detect a 1.2-point difference on the VAS before and after treatment, with a 0.05 probability of type I error. To determine the sample size, we used the results of a previous pilot study (25). In this study, a mean reduction of 2.07 in the acupuncture group and 0.9 in the comparison group was reported. The required sample size was 15 patients in each group (acupuncture and fluoxetine). The analysis, which continued up to 8 weeks after randomization, was based on an intention-totreat principle; this means that all randomized patients were included in the analysis, and they were analyzed according to the randomized treatments.

We used Wilcoxon rank-sum tests for within-group comparisons and Mann-Whitney U tests for between-group analysis of VAS and TPN. VAS and TPN values were analyzed using median and range. For categorical variables, we used either a χ^2^ test or Fisher’s exact test. Standard statistical software package SPSS 13.0 for Windows was used for the analyses. All reported p-values were 2-tailed and considered significant at the 0.05 level.

## Results

All of our patients were females aged 25–65 years (Table 1).

### Primary outcome measure

At baseline, the two groups were statically equal for VAS. At 2-weeks, the median VAS in the group that received acupuncture was 5.0 compared with 8.0 in the group receiving fluoxetine. This difference was statistically significant. VAS evaluations at 4 and 8 weeks of follow-up were not statistically different between the 2 groups (Table 2).

**Table 2:** Results of VAS and TPN. Values are reported as median (range)

Variable	Acupuncture	Fluoxetine (comparison)	P-Value
Baseline (T0)	n = 15	n = 15	
VAS	8.0 (4.0-10.0)	8.0 (4-10)	
TPN	17.0 (11-18)	16.5 (11-18)	
2 weeks (T1)	n = 15	n = 15	
VAS	5.0 (0.0-10.0)	8.0 (4.0-7.0 )	<0.001 a *
TPN	12.5 (3-18)	17.0 (7-18)	<0.001 a *
4 weeks (T2)	n = 15	n = 15	
VAS	7.0 (2.0-10.0)	7.5 (3.0-10.0)	0.18 a
TPN	14.0 (3-18)	16.0 (10-18)	0.016 a *
8 weeks (T3)	n = 15	n = 15	
VAS	7.0 (0.0-10.0)	7.0 (3.0-10.0)	0.65 a
TPN	15.0 (5-18)	15.0 (12-18)	0.47
12 months (T4)	n = 15	n = 15	
VAS	7.0 (0.0-10.0)	8.0 (2.0-10.0)	0.58 a
TPN	15.0 (6-18)	16.0 (7-18)	0.16 a

^*^statistically significant. VAS: visual analogue scale; TPN: number of tender points

^a^Mann Whitney U test

### Secondary outcome measure

Mean TPN in the acupuncture group was 12.8 at baseline, 7.7 at the 2^nd^ week, 6.7 at the 4^th^ week, and 7.2 at the 8^th^ week of follow up. Mean TPN in the control group was 13.6 at baseline, 13.4 at the 2^nd^ week, 12.3 at the 4^th^ week, and 11.6 at 8^th^ week of follow up. Comparison of TPN between these weeks of follow up in acupuncture group demonstrated a significant decrease in TPN with time. In fluxetine group, there was no significant decreased of TPN over time.

Table 3 presents the results of the repeated-measures analysis of variance for the FIQ. This analysis revealed a positive group effect of acupuncture that was statistically superior to the fluxetine group. Subscale analysis revealed significant group effects for symptoms of fatigue and anxiety. The remainder of the subscales also showed trends toward improvement of symptoms, although they were not statistically significant individually.

**Table 3:** Analysis of Variance for the Fibromyalgia Impact Questionnaire (FIQ)*

	Group effect to 8 weeks		Group effect to 4 weeks	
FIQ scale	Acupuncture-fluoxetine mean estimate (95% CI)	P value	Acupuncture-fluoxetine mean estimate (95% CI)	P value
Total	–4.3 (–7.7 to –0.9)	0.02	–4.9 (–8.7 to –1.2)	0.01
Physicalfunction	–0.3 (–0.9 to 0.3)	0.27	–0.4 (–1.1 to 0.3)	0.28
Well-being	+0.4 (–0.6 to 1.4)	0.41	+0.8 (–0.4 to 2.0)	0.18
Pain	–0.7 (–1.5 to 0.3)	0.07	–0.8 (–1.8 to 0.2)	0.14
Fatigue	–0.9 (–1.6 to –0.2)	0.02	–1.2 (–2.1 to –0.4)	0.007
Sleep	–0.3 (–1.3 to 0.6)	0.49	–0.7 (–1.8 to 0.5)	0.25
Stiffness	–0.6 (–1.6 to 0.4)	0.26	–1.0 (–2.3 to 0.3)	0.16
Anxiety	–1.1 (–1.9 to –0.2)	0.02	–1.1 (–2.0 to –0.2)	0.02
Depression	–0.7 (–1.6 to 0.2)	0.14	–0.7 (–1.6 to 0.3)	0.18

^*^Acupuncture- comparison mean estimate is derived from a repeated-measures analysis of variance model. This value is the mean expected difference between acupuncture and fluoxetine group with respect to the particular FIQ subscale, adjusted for time (days since baseline measurement) and baseline subscale value. Negative values for this estimate indicate that values for the acupuncture are lower than the fluoxetine group. Positive values indicate that values for the acupuncture are higher. The P values test whether the group effect is significantly different from 0. P<0.05 suggests a difference between treatment groups with respect to the particular FIQ subscale. CI = confidence interval

**Figure 1:**
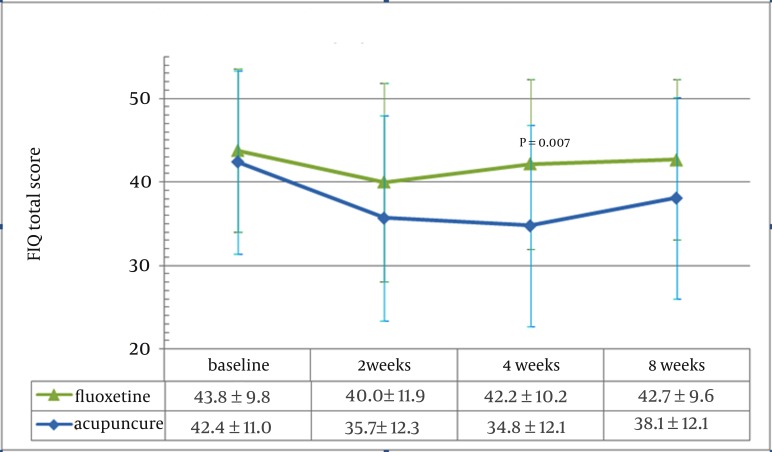
Effect of acupuncture and comparison on the fibromyalgia impact Questionnaire (FIQ)total score

Figure 1 shows the difference in FIQ score between the acupuncture and fluoxetine receivers at each time point, with the greatest difference at 4 weeks (P=0.007). More detailed comparisons of the FIQ measurements at each time point are displayed in Table 4. The total FIQ score in the acupuncture group was improved 7.6 points over the comparison group at 4 weeks after treatment. Other symptoms that showed a statistically significant improvement included fatigue, anxiety, and affective distress. All symptom subscales showed some improvement, although not all of them were statistically significant.

**Table 4:** Results of the Fibromyalgia Impact Questionnaire (FIQ)*

Symptoms	Baseline			2 weeks after treatment			4 weeks after treatment			8 weeks after treatment		
	Acupunc-ture	Fluoxetine	P value	Acupunc-ture	Fluoxetine	P value	Acupunc-ture	Fluoxetine	P value	Acupunc-ture	Fluoxetine	P value
Total score	42.4±11.0	43.8±9.8	0.60	35.7±12.3	40.0±11.9	0.28	34.8±12.1	42.2±10.2	0.007	38.1±12.1	42.7±9.6	0.24
Physical impairment	4.1±2.4	2.7±2.0	0.45	3.2±2.2	3.6±2.5	0.11	3.7±2.5	3.3±2.3	0.96	3.5±2.5	3.3±2.2	0.85
Feel good	3.3±2.7	6.5±1.8	0.40	4.3±2.9	3.6±2.9	0.58	4.6±2.9	3.1±2.4	0.10	3.8±2.9	3.6±2.3	0.99
Pain	6.2±2.2	7.6±1.8	0.63	4.9±2.6	5.6±2.3	0.42	4.7±2.4	5.9v2.3	0.09	5.5±2.3	6.4±2.1	0.25
Fatigue	7.6±2.1	7.3±2.4	>0.99	6.5±2.6	7.1±2.5	0.39	5.6±2.7	7.7±2.1	0.001	7.0±2.4	7.6±1.9	0.34
Rest	6.9±2.1	6.8±2.0	0.53	5.6±2.9	6.4±3.0	0.39	5.9±3.1	6.8±2.2	0.28	6.1±2.9	6.3±2.5	0.89
Stiffness	7.2±1.9	5.5±2.2	0.52	5.6±3.0	6.0±2.8	0.49	5.8±2.7	6.6±2.9	0.11	6.5±2.7	6.8±1.9	0.61
Anxiety	4.2±2.9	4.0±3.1	0.09	3.3±2.7	4.3±2.8	0.79	2.6±2.3	5.1±2.6	0.003	3.3±2.7	4.8±3.0	0.31
Depression	2.9±3.0	4.0±3.1	0.21	2.4±2.8	3.4±3.1	0.90	2.0±2.4	3.7±2.7	0.6	2.2±2.6	3.6±3.1	0.29

^*^Data are preseented as mean ± 1 SD from anaalysis of covariaance, adjusted ffor baseline vallues

## Discussion

The aim of this study was to evaluate the benefit of acupuncture compared with fluoxetine in patients with fibromyalgia. Results showed that acupuncture could improve all pain outcome measures (VAS and TPN) in short-term follow-up; for intermediate-term follow-up after acupuncture treatment, pain measured by TPN was also reduced. At both follow-up intervals, there was improvement noted in the TPN. Our results, however, did not demonstrate benefits at the longer term follow-up in any of the pain outcome measures.

Patients’ VAS and TPN scores in the acupuncture group also showed improvement compared with their pre-treatment values and all follow-up evaluations. It was interesting to find that patients with such a severe disease improved if they received acupuncture.

Our study also demonstrated that, at short-term follow up, 88% of the patients in the acupuncture group experienced a relevant improvement in pain intensity after 2 weeks of acupuncture and 79% of them showed improvement in their TPN score. After 1 year,this improvement dropped to 62 and 61%, respectively.

Systematic reviews looking at the effectiveness of acupuncture for fibromyalgia have found a limited number of high-quality scientific studies. There is moderate evidence that acupuncture is more effective than sham acupuncture in improving symptoms of fibromyalgia (26-28). The quality of the randomized trials in this area has been criticized because of small study populations and short duration of follow-up intervals (29). However, a recently published randomized trial demonstrated that acupuncture was no better than three different forms of sham acupuncture in relieving subjective pain among patients with fibromyalgia (30). There is a need for acupuncture trials that include a large sample size of patients with similar and well-defined pain conditions. Such trials should utilize standardized and well-described acupuncture stimuli. These should be placed effectively in accordance with classical Chinese experience and utilize unequivocal primary outcome measures for the effect (31). Several previous randomized (32-38) and nonrandomized (39,40) trials have demonstrated a beneficial effect of acupuncture in fibromyalgia patients. The only contradictory result was found in a study by Assefi et al (30), who could not demonstrate any benefit of acupuncture in relieving pain compared with three different types of sham treatment. One explanation may be that Assefi et al. did not use any adjunctive therapies with acupuncture to treat the patients. Cassisi et al (41). also demonstrated benefits when acupuncture was combined with antidepressants. Dosage of the SSRI antidepressant used by our patients was according to the routine medical treatment of our pain clinic and was kept the same throughout the study. A usual-care comparison group, like the one used in our pain clinic, has not been described in the literature until now. So, we decided to evaluate the effect of acupuncture on pain compared with drug treatment by fluoxetine. Therefore, acupuncture was evaluated as a part of the medical treatment of fibromyalgia. We understand that, due to the complexity of fibromyalgia syndrome, associated with the severity of symptoms, monotherapy modalities are not indicated. Similar to other randomized controlled trials that evaluated the effect of acupuncture in fibromyalgia patients (30,37); we made a potentially controversial decision to standardize the acupuncture points in order to allow reproducibility of the technique and to help us to assess the efficacy of a specific acupuncture procedure. We realize that the use of acupuncture at fixed points may differ from acupuncture that is practiced in clinical settings where therapy is usually individualized (30,33).

We agree with Assefi et al (30) in that there is no consistent data suggesting that individualized acupuncture is superior to fixed-point prescriptions. Also, no gold standard exists for the selection of acupuncture points for treatment of fibromyalgia. In the absence of data, similarly to Assefi et al. we designed a protocol based on our clinical experience. We selected acupuncture points that could provide symptomatic relief in our patients, since the pathogenesis and etiology of fibromyalgia remains unknown. Our study reported similar results to those by Martin et al (37). Who found that acupuncture was effective to provide significant symptomatic relief in patients with fibromyalgia in short-term but not long-term. We found that acupuncture improved symptoms of fibromyalgia significantly more than fluoxetine. All symptom subscales were improved with acupuncture, but only fatigue and anxiety were statistically significant on their own. The pain trend was closely toward statistical significance in the FIQ. However, fibromyalgia is a syndrome of symptoms not just pain. Similar to Martin et al (37). our patients were homogenous in diagnosis, characteristics and severity of symptoms. The Fibromyalgia Treatment Program has been shown to reduce the mean FIQ total score from 51.3 to 44.7 (42). The average FIQ total score of our patients after the Fibromyalgia Treatment Program was 42.5, which is close to the expected value. The improvement observed in our study was additive to the benefits obtained with the Fibromyalgia Treatment Program (i.e., educational and behavioral interventions).

We saw maximum benefit at 4 weeks (among time points we considered), and that benefit was less significant at 8 weeks. The time course of improvement after acupuncture should be better characterized in future studies. Our study showed that acupuncture reduced the FIQ score by 7 points. This benefit was additive to the beneficial effect produced by the Fibromyalgia Treatment Program, which also produced a mean benefit of 7 points (42). The magnitude of clinical benefit produced by acupuncture is similar to that reported with pharmacological interventions (43,44). Therefore, the effect of acupuncture is both clinically and statistically significant. There is evidence that the effects of acupuncture may last for years after a course of treatment. Waylonis (40) also found that the benefit lasted from one month to one year after treatment. Others, such as Deluze et al (36). did not present any long-term results. In fact, their patients were only evaluated before the first acupuncture session and after that treatment. The other two high-quality randomized controlled trials only followed patients up to 6 months (30) and 7 months (37) after completion of their treatment. In our study, we followed patients up to 1 year. Our results, however, did not demonstrate any longer term benefit of acupuncture in the pain outcome measures.

One advantage of acupuncture treatment is that it produces fewer adverse side-effects compared with many drugs used to treat fibromyalgia symptoms (21). McCartney et al (45). similarly reported a case of bilateral hand edema after using bilateral LI-4 acupoints to treat chronic low-back pain and sciatica. Similar to Assefi et al (30), Martin et al (37) and Costa (38), acupuncture treatments were also very well tolerated by our patients. Our patients did not report discomfort, soreness, vasovagal symptoms, bruising or hematomas during the treatment period, and did not report other complaints during the follow-up evaluations.

Acupuncture in FMS can be used simultaneously with other alternative medicines (46) and acupuncture combined with western medicine (47,48) or use of physical modalities as laser irradiation on acupoints (48) may be superior to acupuncture unaccompanied.We feel that adding acupuncture to standard care reduces the pain of fibromyalgia, especially when the cost of this treatment is weighed against the costs of this painful chronic condition, all of which must be borne by patients, their families and by society in general.

According to our primary outcome measure (VAS), acupuncture was more effective than treatment with fluoxetine. TPN in the acupuncture group also showed improvement compared with their pre-treatment values. Total fibromyalgia symptoms, as measured by the FIQ, were significantly improved in the acupuncture group compared with the control one. As result of above, symptoms of FMS improved with acupuncture both clinically and statically. Acupuncture has no any complication or side effect on our patients and duration of treatment was shorter (2 week) than fluoxetine (8 weeks). Acupuncture is also cost effective. Needling of acupuncture is not painful and patients’ fear of needling is only before starting the treatment and after the first session of treatment no any patient has fear and anxiety of needling.

## Conclusion

Our study was a prospective randomized clinical trial comparing acupuncture with fluoxetine in fibromyalgia. In conclusion, acupuncture was effective, well tolerated and without adverse effect treatment for fibromyalgia. Application of acupuncture as a usual treatment for FMS may be beneficial for pain reduction and other symptoms of disease.
